# The number of nephrons in different glomerular diseases

**DOI:** 10.7717/peerj.7640

**Published:** 2019-09-04

**Authors:** Davide Viggiano, Michelangelo Nigro, Francesco Sessa, Graziano Vignolini, Riccardo Campi, Sergio Serni, Rosa Maria Pollastro, Gianfranco Vallone, Giuseppe Gigliotti, Giovambattista Capasso

**Affiliations:** 1 Department of Medicine and Health Sciences, University of Molise, Campobasso, Italy; 2 UOC of Nephrology and dialysis, Eboli Hospital “Maria SS Addolorata”, Eboli, Italy; 3 Department of Urologic Robotic Surgery and Renal Transplantation, University of Florence, Careggi Hospital, Florence, Italy; 4 Department of Experimental and Clinical Medicine, University of Florence, Florence, Italy; 5 Department of Translational Medicine, University of Campania “L. Vanvitelli”, Naples, Italy; 6 Department of Radiology, University of Naples “Federico II”, Naples, Italy; 7 Biogem, Ariano Irpino, Italy

**Keywords:** Nephron number, eGFR, Blood pressure, Nephrotic syndrome, Nephritic syndrome, IgA nephropathy, Diabetic nephropathy, Membranous nephropathy, Minimal change disease, Podocytes

## Abstract

**Background:**

The total number of nephrons has been measured mainly from post-mortem studies and only in selected populations. Data from living subjects are scanty, and direct comparisons among different glomerular diseases are lacking. The present work exploits modern methodology to estimate the total nephron number in glomerulopathies with prevalent proteinuria/nephrotic syndrome versus glomerulopathies with nephritic syndrome (IgA nephropathy (IgAN), lupus nephritis), thus extending previous observations about the number and function of glomeruli in different physiological and pathological states.

**Methods:**

This is a retrospective study based on one hundred and seven patients who have undergone renal biopsy. The glomerular density has been estimated from the biopsy specimens and the total cortical volume has been obtained from ultrasound recordings. Stereological methods have been applied to calculate the total number of nephrons and their volume. The correlation between clinical parameters and quantitative morphological data have studied using the Pearson correlation coefficient (*r*).

**Results:**

The total number of nephrons inversely correlated with the systolic blood pressure (*r* = −0.4, *p* < 0.05). In proteinuric diseases, such as focal segmental glomerulo-sclerosis (FSGS), membranous nephropathy (MN) and diabetes, the change in estimated GFR (eGFR) directly correlated with the total number of non-sclerotic glomeruli (NSG) (*r* = 0.62, *p* < 0.01), whereas in nephritic syndrome no significant correlation was observed. The alterations in eGFR occurring in nephritic syndromes such as IgAN cannot be explained on the basis of the number of NSG.

**Discussion:**

The fusion of the podocyte foot-processes that typically occurs in purely proteinuric diseases does not modify the glomerular filtration rate: therefore in these situations, the change in eGFR depends mainly on the number of available glomeruli. On the other side, the eGFR decrease occurring in nephritic syndromes, such as IgAN, cannot be explained simply on the basis of the number of NSG and likely depends on the substantial involvement of the mesangial axis. Future studies should verify whether these changes are reversible with appropriate therapy, thus reversing eGFR decrease.

## Introduction

According to the “intact nephron hypothesis” proposed by Neal Bricker in 1960, a greater fraction of the total renal excretion must be performed by fewer, functionally intact tubules when kidney damage occurs (Bricker, Morrin & Kime, 1960; Hayman et al., 1939; Platt, 1952). However, in 1974 the work by Barry Brenner’s laboratory in animal models (Deen et al., 1974) emphasized that also the remaining glomeruli increase their filtration rate (GFR; “hyperfiltration”) and become hypertrophic. The increase in the GFR would be a maladaptive response that may damage the glomeruli (Hostetter et al., 1981). In 1986–1993, Barker proposed that an abnormal fetal environment (low-birth weight) can favor older age pathologies (e.g. coronary heart disease and hypertension) (Barker & Osmond, 1986, 1988; Barker et al., 1993). These hypotheses were then merged into the “nephron under-dosing” (Brenner, Garcia & Anderson, 1988): an inherited, low nephron endowment (e.g. in preterm infants) would lead to subclinical hyperfiltration and greater risk of hypertension in chronic kidney disease (CKD) (Brenner, Lawler & Mackenzie, 1996). The anatomical correlate of the hyperfiltration is thought to be an increase in the glomerular volume (Fogo & Ichikawa, 1991). Accordingly, in autopsy studies, a correlation between the total number of glomeruli and mean glomerular volume has been repeatedly observed (Hoy et al., 2003; Hughson et al., 2008), suggesting that a lower number of nephrons is compensated by glomerular hypertrophy. The recent data by Denic et al. (2017a) confirm these observations in vivo, as a higher single-nephron measured glomerular filtration rate is associated with larger nephrons and more glomerulosclerosis. Unfortunately, autopsy data gave contrasting information regarding a reduced number of nephrons in hypertensive subjects (Hughson et al., 2006, 2014; Kanzaki et al., 2017).

Several systemic diseases such as diabetes (Bank, 1991) and obesity (Chagnac et al., 2000) are initially accompanied by hyperfiltration, and at later stages by hypofiltration (Anastasio et al., 2017). It is unclear if this also applies to CKD (Brenner, Lawler & Mackenzie, 1996; Kanzaki et al., 2017). Accordingly, obesity and diabetes (Tsuboi et al., 2012), CKD (Kanzaki et al., 2017), minimal change disease (MCD) (Koike et al., 2011), focal segmental glomerulosclerosis (FSGS), membranous nephropathy (MN) (Tsuboi et al., 2011) and IgA nephropathy (IgAN) (Tsuboi et al., 2010) lead to a reduced number of nephrons. In these pathologies the presence of larger, hypertrophic glomeruli has also been observed: hypertension, diabetes, obesity (Tsuboi et al., 2012), CKD, MCD (Fogo et al., 1990), FSGS (Nishimoto et al., 1997) and IgAN (Tóth & Takebayashi, 1998). The glomerular hypertrophy in early obesity and hypertension might, however, derive from a greater metabolic and excretory demand (Osterby & Gundersen, 1975).

Therefore, the reduction of the estimated GFR (eGFR) in kidney diseases would result in part from a reduced number of non-sclerosed glomeruli (the “chronic” part of the disease) and a possibly reversible, functional hypofiltration at the level of individual glomeruli (the “acute” part of the disease). Empiric observations have validated the relation between the number of non-sclerosed glomeruli and the eGFR (Kanzaki et al., 2017).

Several proteinuric glomerular diseases (e.g. diabetic nephropathy (DN), FSGS, MCD, amyloidosis) show damages of the podocytes in terms of loss of foot processes commonly referred to as “effacement” (Shankland, 2006) and thought to be responsible for the nephrotic syndrome. The effacement of foot process is much less evident in nephritic syndromes (with hematuria and low proteinuria) such as IgAN (van den berg et al., 2004). The different behavior of podocyte foot process in these two conditions (nephrotic/proteinuric vs nephritic syndromes) leads to speculation that the change in eGFR has different etiology in these conditions. In this work we compared the association between nephron number and eGFR in nephrotic and nephritic syndromes. In other terms, we expect that the relationship GFR-nephron number has a different slope in proteinuric syndromes compared to nephritic syndromes. Although several data are now available about the total number of nephrons, data regarding CKD patients with nephritic and nephrotic syndromes are still scanty, and this work aims at filling this knowledge gap.

## Materials and Methods

### Study population

This is a retrospective, observational cross-sectional study. After Institutional Review Board approval and informed consent, data in the period 2014–2018 from patients were retrospectively collected in an a priori developed dataset. Data were taken from the clinical and immune-pathological records of the Nephrology Unit of the Eboli Hospital, the Nephrology Unit of the University of Campania “L. Vanvitelli” and the Department of Urological Robotic Surgery and Renal Transplantation, University of Florence, Careggi Hospital, Florence. Written informed consent was obtained from the participants.

One hundred and seven renal biopsies were analyzed in this pilot study. The following inclusion criteria were used: (1) histological diagnosis of FSGS, MN, DN, MCD, IgMN, IgAN, Lupus nephritis; (2) age between 20 and 60 years. Furthermore, patients in the proteinuric group were excluded if proteinuria was less than 1g/24 h.

Of the initially identified 107 patients, only 59 satisfied the inclusion criteria, and were included in further analysis.

The diagnosis of the subjects derived from histological and immunofluorescence (using standard panels of antibodies) staining of tissue biopsies. Only biopsies containing at least four glomeruli were used. The minimum number of glomeruli to select reliable biopsies was based on previous evidence (Denic et al., 2017a, 2017b). In preliminary study, we found no significant correlation between the area of the section (that is the size of the biopsy sample) and the measured variables.

### Kidney function and clinical characteristics

Clinical parameters (age, gender, body weight, systolic and diastolic arterial blood pressure) and hematological parameters (calibrated creatinine, blood urea nitrogen and uric acid) have been retrieved for each patient before the biopsy procedure, and the GFR was estimated using the CKD-EPI formula. Subject height was not available from clinical recordings, therefore the BMI could not be calculated and only the body weight was included in the analysis. Hypertension was defined as blood pressure ≥140/90 mmHg.

### Imaging

Ultrasound images in longitudinal and transversal planes were also analyzed for each patient. For each center, only one ultrasound operator acquired the kidney images. The protocol for image acquisition was the same in the three centers. Three different ultrasound devices were used in the three centers. We could not detect systematic differences in ultrasound parameters among centers. In 35 patients the kidney volume estimated by ultrasound was also compared to the kidney volume measured using the Cavalieri principle on CT scan images, confirming the absence of bias among centers in the estimate of kidney volume.

### Biopsy morphology

The biopsy specimens have been fixed in Bouin, processed and embedded in paraffin using routine methods. Two and half micron-thick sections (2.5 µ) were stained with hematoxylin-eosin or periodic acid schiff. The entire sections have been scanned using an Aperio CS2 scanner (Leica): the use of this system of whole slide image, allows viewing virtually the entire biopsy at high resolution (0.8 pixel per micron).

Section images have been analyzed using the ImageJ free software. The total number of glomeruli, the number of non-sclerotic glomeruli (NSG) per unit area of cortex (mm^2^) was manually counted:

$$\displaylines{
  {\rm{ADglo}}{{\rm{m}}_{{\rm{NSG}}}}\;({\rm{n/m}}{{\rm{m}}^2}) = {\rm{NSG\, Area\, Density}} \hfill \cr 
  \qquad\qquad\qquad\qquad\,\,\,\ \,\,\,\,   = {\rm{number\, of\, NSG/area\, of\, cortex\, in\, the\, specimen\, }}({\rm{m}}{{\rm{m}}^{\rm{2}}}) \cr} $$

The percent of NSG was calculated as: 100 × number of NSG/total number of glomeruli.

The area covered by each glomerulus was also measured and the proportional area of NSG (PrAglom_NSG_ = total area of NSG/area of cortex) was calculated. The area considered was only the region of the histological section containing the glomeruli, excluding the medulla that could be present in the section.

Interstitial fibrosis/tubular atrophy, that is the percentage of cortical area involved by the tubular atrophy or interstitial fibrosis, was defined as follows: T0, 0–25%; T1, 26–50% and T2, >50%.

The reliability of the measurements has been tested by repeating the analysis by a second observer.

### Estimation of kidney volume and parenchymal volume

The total kidney volume (including the renal sinus) was estimated from ultrasound images using the following ellipsoid-KV3 formula proposed by Higashihara et al. (2015), which had better performance than the ellipsoid formula:

$${\rm{Total\, kidney\, volume}} = 84\; + \;1.01\; \times \;\pi /24\; \times \;{\rm{LD}}\; \times \;{({\rm{ML}} + {\rm{AP}})^2}$$

where LD (longitudinal diameter) is the maximum axis of the kidney in longitudinal plane, ML is the medio-lateral axis and AP the antero-posterior axis, both perpendicular to LD and passing through its midpoint (see Fig. S1).

The volume of the renal sinus was also estimated using the same approach. The parenchimal volume was established as the difference between the total kidney volume and the volume of the renal sinus. The volume of the kidney cortex was then estimated using a constant cortex/parenchyma ratio of 0.7 (Denic et al., 2017a, 2017b, Wang et al., 2014).

To verify the bias of this approach, a pilot study using CT images with contrast medium from 35 patients identified the cortical volume using the Cavalieri principle; the coefficient of correlation between the true parenchymal volume and the estimate using the ellipsoid was 0.8 (*p* < 0.01). Similarly, the coefficient of correlation between the true parenchymal volume and the estimate using the ellipsoid-KV3 was 0.8 (*p* < 0.01).

Furthermore, we also estimated the cortical volume using the formula by Nakazato, Ikehira & Imasawa (2017) as previously described.

$$\eqalign{
  & {\rm{Estimated\, volume\, of\, the\, renal\, cortex\, }}({\rm{c}}{{\rm{m}}^3})  \cr 
  & \quad {\kern 1pt}  = 0.012\; \times \;{\rm{renal\, length\, }}{({\rm{cm}})^{0.92}}\; \times \;{\rm{width\, }}{({\rm{cm}})^{0.53}}\; \times \;{\rm{body\, weight\, }}{({\rm{kg}})^{0.40}}  \cr 
  & \quad \quad \; \times \;{\rm{body\, height\, }}{({\rm{cm}})^{0.67}}\; \times \;{\rm{eGFR}}\;{({\rm{ml/min/}}1.73\;{{\rm{m}}^2})^{0.22}}\; \times \;1.12\;{\rm{if\, diabetes\,}}{\rm{.}} \cr} $$

The Pearson coefficient of correlation between the cortical volume and that estimated using the Nakazato formula was 0.62 (*p* < 0.01).

### Estimation of total number of nephrons

The estimate of number of nephrons per kidney follows the method reported by Denic et al. (2017a, 2017b).

Briefly, first the volume of NSG (VglomNSG) and the NSG volume density (DglomNSG, n/mm^3^ of cortex) were estimated by the Weibel–Gomez (Weibel & Gomez, 1962) stereological model, allowing to estimate three-dimensional information (volume, counts/volume) from two-dimensional biopsy images:

$$\eqalign{
  & {\rm{Vglo}}{{\rm{m}}_{{\rm{NSG}}}} = {\rm{NSG\, volume \,}}({\rm{m}}{{\rm{m}}^3}) = 1.382\; \times \;{({\rm{Mean\, area\, of\, NSG}})^{1.5}}/1.01  \cr 
  & {\rm{Dglo}}{{\rm{m}}_{{\rm{NSG}}}} = {\rm{NSG\, volume\, density\, }}({\rm{n/m}}{{\rm{m}}^3})  \cr 
  & \quad \quad \quad \quad \; \ = 0.724\; \times \;{[{({\rm{ADglo}}{{\rm{m}}_{{\rm{NSG}}}})^3}/({\rm{PrAglo}}{{\rm{m}}_{{\rm{NSG}}}})]^{0.5}} \cr} $$

The total number of nephrons per kidney was therefore:

$$\eqalign{
  & {\rm{Nglo}}{{\rm{m}}_{{\rm{NSG}}}} = {\rm{total\, number\, of\, nephrons\,}}  \cr 
  & \quad \quad \quad \quad \; \, \, = {\rm{Dglo}}{{\rm{m}}_{{\rm{NSG}}}}\;({\rm{n/m}}{{\rm{m}}^3})\; \times \;{\rm{cortical\, volume\, }}({\rm{m}}{{\rm{m}}^3})/1.81 \cr} $$

The correction factor 1.81 was needed to control for tissue shrinkage of histological sections and volume shrinkage of biopsy core due to loss of blood perfusion (Fulladosa et al., 2003; Lerman et al., 1990).

### Statistical analysis

Statistical analyses were performed using SPSS Statistics 13 for Windows and R environment. Continuous data were summarized and presented as means ± SD. Categorical variables are presented as percentage.

Normality distribution was tested with the Shapiro–Wilk test. Since most of the variables did not satisfy the normality assumption, the Mann–Whitney *U* test was used in place of the *t*-test to compare the two groups (nephrotic vs nephritic patients). For categorical variables (gender), the chi-square statistic was used to compare the two groups.

To correlate the clinical parameters to quantitative morphological data, the Pearson’s test was calculated separately for each variable. The population size was selected in order to find a Pearson’s correlation coefficient (*r*) above 0.4 between clinical variables and morphological data, with a power of 80%. We defined statistical significance as *p* < 0.05.

## Results

The mean value of the parameters of the patients and the density of glomeruli are reported in Table 1. The eGFR was significantly correlated with the age (*r* = −0.31, *p* < 0.01). The total number of glomeruli was only border-line correlated to the patient’s age (*r* = −0.21, *p* = 0.06). The % globally sclerosed glomeruli was not significantly correlated with age in this population with kidney diseases (*r* = 0.07, *p* = 0.56).

**Table 1:** Clinical data of the populations under study (data represent mean ± SEM).

	Proteinuric/Nephrotic syndrome	Nephritic syndrome	*p* (test statistics in parenthesis)
*N* (total = 59)	33 (FSGS = 14; MN = 8; DN = 4; IgMN = 2; MCD = 5)	26 (IgAN = 21; lupus nephritis = 5)	
Gender (F/M)	9/24 (27%/73%)	11/15 (42%/58%)	0.27 (Chi-square test)
Age (years)	48 ± 13	42 ± 16	0.06 (Mann–Whitney *U*)
Body weight (Kg)	80 ± 18	79 ± 16	0.99 (Mann–Whitney *U*)
SBP (mmHg)	130 ± 15	134 ± 15	0.15 (Mann–Whitney *U*)
DBP (mmHg)	78 ± 9	82 ± 10	0.18 (Mann–Whitney *U*)
Prevalence of hypertension	34%	50%	0.38 (Chi-square test)
Urea (mg/dl)	71 ± 33	60 ± 43	0.08 (Mann–Whitney *U*)
Creatinine (mg/dl)	1.8 ± 1.0	1.5 ± 1.3	0.10 (Mann–Whitney *U*)
Uric acid (mg/dl)	6 ± 1.6	5.9 ± 2.0	0.84 (Mann–Whitney *U*)
Proteinuria (mg/24 h)	4,248 ± 3,085	1,340 ± 1,362	<0.01 (Mann–Whitney *U*)
Estimated glomerular filtration rate (ml/min/1.73 m^2^)	56 ± 34	74 ± 35	0.06 (Mann–Whitney *U*)
TA/IF score (T0/T1/T2)	42%/38%/19%	68%/31%/0%	0.08 (Chi-square)
Kidney length (mm)	109 ± 11	106 ± 10	0.25 (Mann–Whitney *U*)
Total kidney volume (ml)	265 ± 53	231 ± 48	0.01 (Mann–Whitney *U*)
Kidney parenchyma volume (ml)	155 ± 41	129 ± 40	0.02 (Mann–Whitney *U*)
Kidney cortical volume (ml)	108 ± 29	90 ± 27	0.02 (Mann–Whitney *U*)
Kidney cortical volume (Nakazato formula; ml)	121 ± 45	118 ± 29	0.83 (Mann–Whitney *U*)
% Sclerotic glomeruli	13 ± 17	7 ± 11	0.20 (Mann–Whitney *U*)
Vglom_NSG_ (mm^3^ × 10^−6^)	6 ± 3.2	4.6 ± 2.5	0.05 (Mann–Whitney *U*)
Nglom_NSG_ (× 1,000)	589 ± 288	619 ± 311	0.80 (Mann–Whitney *U*)
Nglom_NSG_ (× 1,000) using Nakazato formula for cortical volume	667 ± 345	795 ± 361	0.23 (Mann–Whitney *U*)

**Note:**

FSGS, focal segmental glomerulosclerosis; MN:, membranous nephropathy; DN, diabetic nephropathy; IgMN, IgM nephropathy; MCD, minimal change disease; IgAN, IgA nephropathy; IF/TA, interstitial fibrosis/tubular atrophy; SBP/DBP, systolic/diastolic blood pressure.

**Figure 1:**
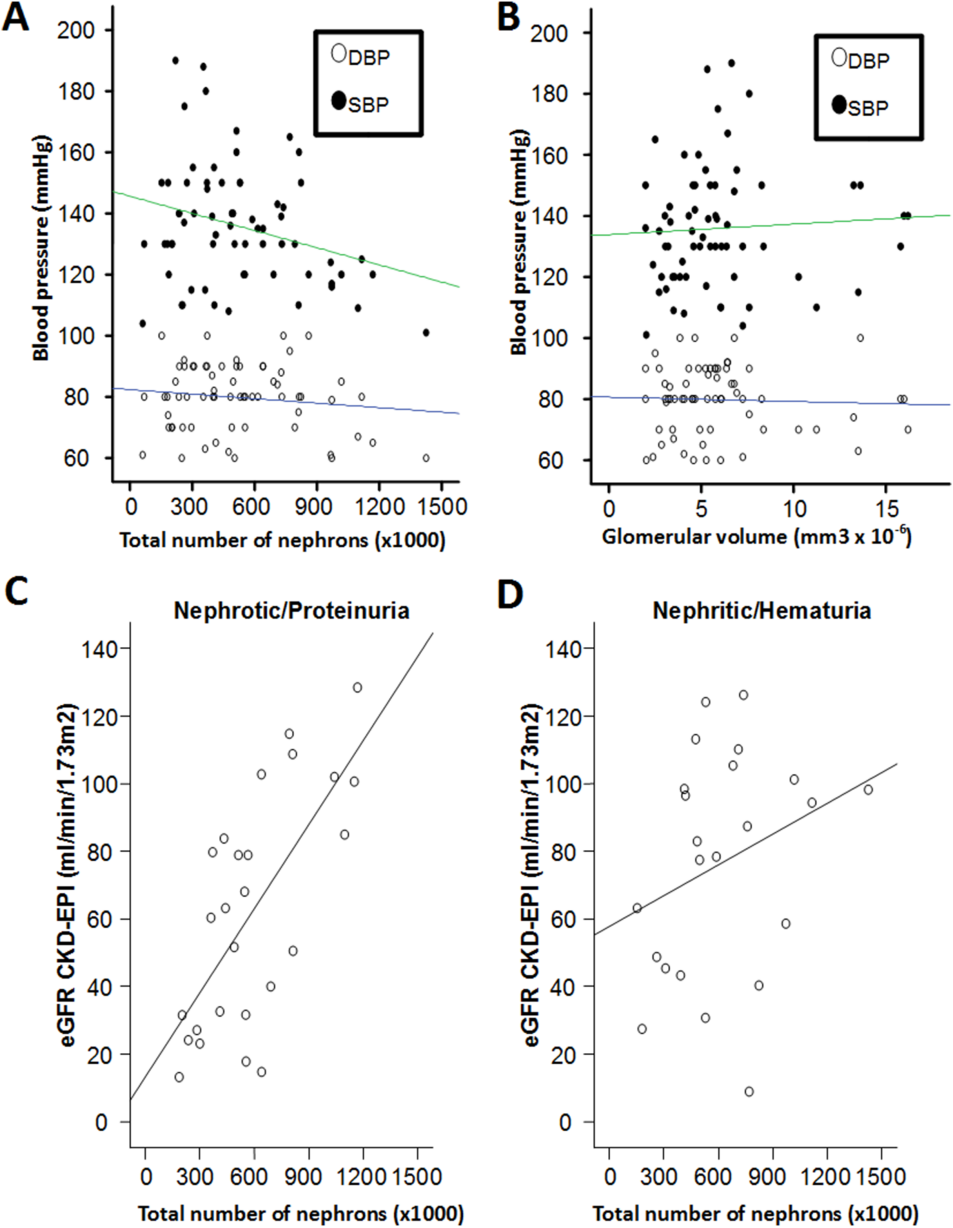
Relationship between total number of nephrons, arterial blood pressure and eGFR. (A) Relationship between total number of nephrons and systolic (SBP, filled circles) and diastolic (DBP, empty circles) blood pressures. (B) Relationship between glomerular volume and systolic (SBP, filled circles) and diastolic (DBP, empty circles) blood pressures. (C–D) Relationship between total number of nephrons and the total eGFR. Only nephrotic/proteinuric syndromes show significant correlation, whereas in nephritic syndromes the two variables are not correlated.

When all data were pooled, the total number of nephrons inversely correlated with the systolic (SBP) blood pressure (SBP: *r* = −0.40, *p* = 0.02; diastolic blood pressure (DBP): *r* = −0.35, *p* = 0.06). This is also reported in Fig. 1A. Conversely, the volume of glomeruli (Vglom_NSG_) did not correlate with the blood pressure (*r* = 0.06 and −0.04, *p* = 0.63 and 0.73 for SBP and DBP respectively, Fig. 1B). We then verified whether the size of glomeruli was correlated with hyperfiltration as indexed by eGFR. When pooling all data, the correlation between eGFR and VglomNSG was not significant (*r* = −0.08, *p* = 0.64). The total number of glomeruli was not correlated with the volume of glomeruli (*r* = −0.22, *p* = 0.18).

There was no significant correlation between nephron number and proteinuria (mg/24 h) (*r* = −0.04, *p* = 0.77).

Finally, higher eGFR correlated with more nephrons (*r* = −0.39, *p* = 0.02).

We therefore tested whether the correlation between the total number of glomeruli and eGFR was different in glomerulopathies accompanied by podocyte foot process effacement (nephrotic/proteinuric syndromes) and in nephritic syndromes (IgAN) (Jameson & Loscalzo, 2017). As reported in Table 1, the two syndromes showed no significant difference in the total number of NSG. However, in nephrotic/proteinuric syndromes the total number of glomeruli predicted the eGFR (*r* = 0.62, *p* = 0.003), whereas in IgAN there was no correlation between eGFR and total number of NSG ( *r* = 0.03, *p* = 0.91). This is also shown in Fig. 1C. Lower eGFR also correlated with older age (*r* = −0.31, *p* < 0.01). The total number of glomeruli was only border-line correlated with the patients age (*r* = −0.21, *p* = 0.06). The % globally sclerosed glomeruli was not significantly correlated with age in this population with kidney diseases (*r* = 0.07, *p* = 0.56).

## Discussion

The total number of nephrons has been measured mainly from post-mortem studies and only in selected populations. Data from living subjects are scanty, and direct comparisons among different glomerular diseases are lacking. The main result of the present study is that the change of eGFR in IgAN is due to a dysfunction of the glomeruli whereas in nephrotic/proteinuric syndromes it reflects the total number of glomeruli (Rauen et al., 2015, 2018). This is the first study in human subjects demonstrating this difference between the two syndromes.

The present work extends previous observations about the number and function of glomeruli in different physiological and pathological states (Tsuboi et al., 2012; Kanzaki et al., 2017; Koike et al., 2011).

The total number of nephrons (NglomNSG) was inversely correlated with the systolic and diastolic blood pressure. This result is consistent with previous reports of a lower number of nephrons in subjects with increased blood pressure (Keller et al., 2003; Kanzaki et al., 2017).

However, the number of nephrons did not correlate with the glomerular volume, in contrast to previous findings (Tsuboi et al., 2010).

The total number of glomeruli predicted the eGFR, as suggested by Kanzaki et al. (2017). However, subgroup analysis revealed that this was true mostly for proteinuric patients. This was consistent with our previous finding that glomerular density correlated with eGFR only in specific subpopulations and not in all glomerular diseases (Nigro et al., 2018). This is consistent also with the hypothesis that the eGFR does not reflect simply the number of nephrons, but also their mean activity, that is GFR depends not only on anatomical but also on functional changes.

Therefore, we believe that the CKD staging does not appropriately reflect the fact that in some diseases a specific stage reflects anatomical changes, whereas in others it may reflect a functional (possibly reversible) problem (Viggiano et al., 2019).

The study explores for the first time the number of nephrons and glomerular volume in different glomerular diseases in vivo. The data are in agreement with previous observations, thus suggesting that major faults in the methodology are not present and that the collateral conclusions of the work are likely correct. Furthermore, it is a multicenter study (Eboli, Naples and Florence). Despite its novelty, our study is not devoid of limitations. First, this is a preliminary experience with small sample size. As such, we could not formally compare the outcomes. Second, ultrasound and other imaging modalities often do not allow for a precise estimate of the kidney cortex, as the cortico-medullary differentiation is very often absent in images. Therefore, our study, in line with other studies, has used a constant cortex-parenchyma ratio. This may lead to a systematic bias (if the ratio is biased). Furthermore, it is far to be proven that the cortex-parenchyma ratio is constant at different eGFR levels: this may have led to further distortion of the total number of nephrons. However, our conclusions are robust even when using the Nakazato formula to estimate the renal cortex, which is not dependent upon an assumption of a constant cortex-parenchyma ratio. Finally, we have used the eGFR formula (CKD-EPI) throughout the analysis. However, the coefficient of variation of the formula greatly increases as the eGFR increases and is very noisy at GFR above 60 ml/min/1.73 m^2^ (though unbiased). Notwithstanding these limitations, the estimate of the total number of nephrons is not very difficult and might become part of routine clinical practice if appropriate measures are routinely presented by the pathologist and the radiologist/ultrasound technician.

To these aims, adequately powered trials with longer follow-up are needed. Overall, larger studies are needed to confirm these findings and to its indications and limits.

## Conclusions

Our data show that in proteinuric diseases the total number of nephrons accounts for most of the variability of eGFR in proteinuric diseases. Therefore, the fusion of the podocyte foot-processes does not modify the glomerular filtration rate. Conversely, in nephritic syndromes the total number of nephrons does not explain the modifications of eGFR. Therefore, in nephritic syndromes the change of eGFR likely depends on the substantial involvement of the mesangial axis. Future studies should verify whether these changes are reversible with appropriate therapy, thus leading to improved eGFR.

## Supplemental Information

10.7717/peerj.7640/supp-1 Supplemental Information 1 Example calculation of kidney volume and parenchymal volume using different methods. (A) Volume estimate from CT scan with Cavalieri’s principle. The area of the total kidney volume and the kidney parenchyma are identified by manual segmentation. The kidney cortex is identified using manual thresholding on the arteriographic phase of CT scan with contrast medium. The sum of the area from all sections is then multiplied by the distance between the sections. (B) Volume estimates from ultrasound images taken on longitudinal and transversal planes. The maximum diameter of the kidney (LD), and the diameters perpendicular to this axis on a transverse plane (AP, ML) are measured, together with the same diameters in the renal sinus (LDs, APs, MLs). The kidney is then approximated to an ellipsoid and the total volume and renal sinus volume calculated. The Ellipsoid-KV3 formula has a correction which improves the estimates. The volume of the parenchyma is estimated as the difference between the total volume and the renal sinus volume. The cortical volume is then calculated from the parenchymal volume using a constant 0.7 correction factor.

10.7717/peerj.7640/supp-2 Supplemental Information 2 Comparison of kidney volume, parenchymal volume and cortical volume using different measurement methods. Data represent mean ± SD from 35 patients with CT scan available.

10.7717/peerj.7640/supp-3 Supplemental Information 3 Raw data used for statistical analysis.

10.7717/peerj.7640/supp-4 Supplemental Information 4 Codebook to convert numbers to their respective factors.

## Additional Information and Declarations

### Data Availability

The following information was supplied regarding data availability: The raw measurements are available in the Supplemental File.
