# Supplementary material for: The number of nephrons in different glomerular diseases
Source: PeerJ. 2019 Sep 4;7:e7640. doi: 10.7717/peerj.7640 (PMC6731770; doi:10.7717/peerj.7640)
Supplement: Supplemental Information 1 — (A) Volume estimate from CT scan with Cavalieri’s principle. The area of the total kidney volume and the kidney parenchyma are identified by manual segmentation. The kidney cortex is identified using manual thresholding on the arteriographic phase of CT scan with contrast medium. The sum of the area from all sections is then multiplied by the distance between the sections. (B) Volume estimates from ultrasound images taken on longitudinal and transversal planes. The maximum diameter of the kidney (LD), and the diameters perpendicular to this axis on a transverse plane (AP, ML) are measured, together with the same diameters in the renal sinus (LDs, APs, MLs). The kidney is then approximated to an ellipsoid and the total volume and renal sinus volume calculated. The Ellipsoid-KV3 formula has a correction which improves the estimates. The volume of the parenchyma is estimated as the difference between the total volume and the renal sinus volume. The cortical volume is then calculated from the parenchymal volume using a constant 0.7 correction factor. [file peerj-07-7640-s001.pdf]

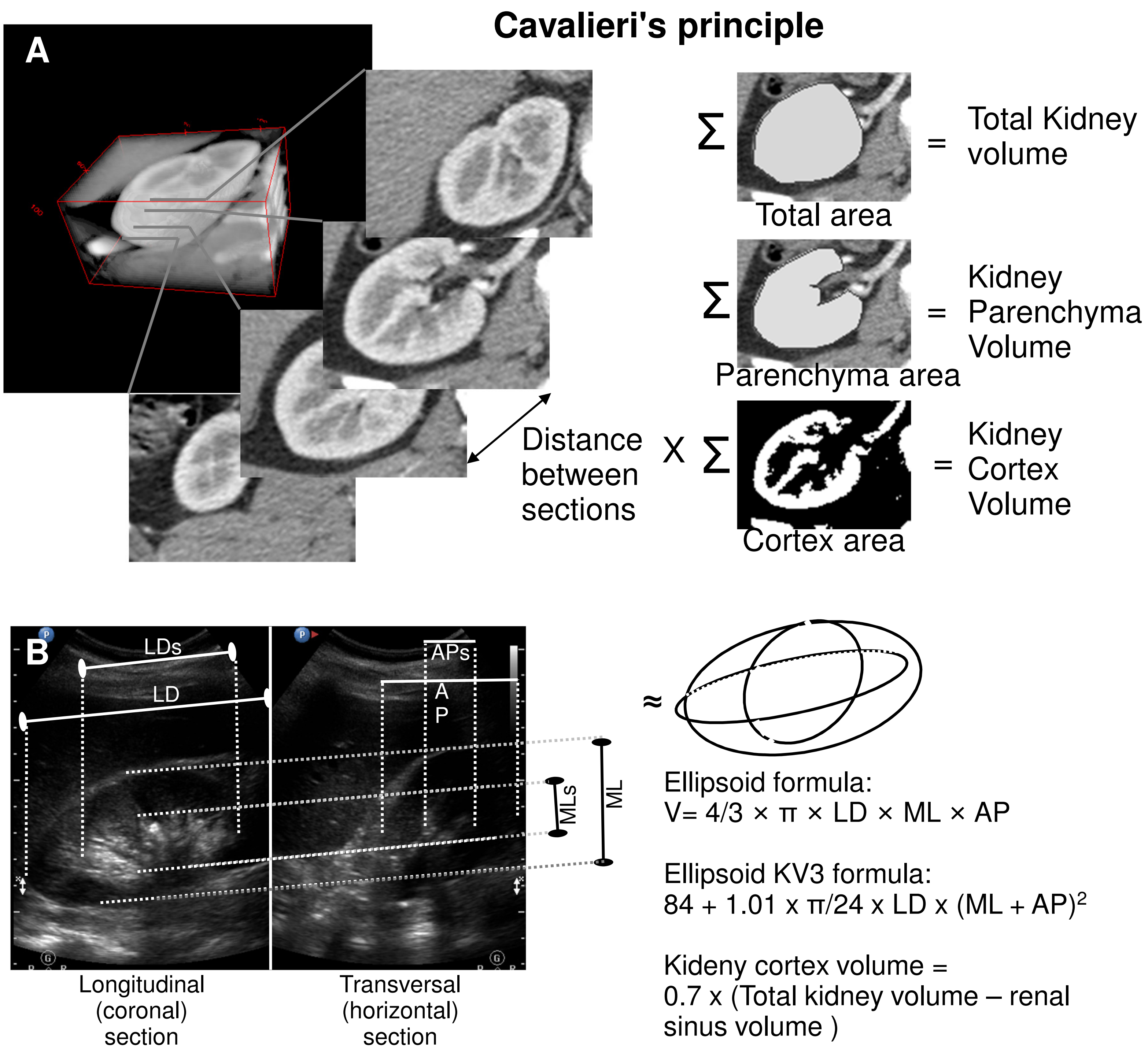

Cavalieri's principle
A
Σ
Total area
= Total Kidney volume
Σ
Parenchyma area
= Kidney Parenchyma Volume
Distance between sections
X Σ
Cortex area
= Kidney Cortex Volume
B
LDs
LD
APs
A
P
MLs
ML
Longitudinal (coronal) section
Transversal (horizontal) section
≈
Ellipsoid formula:
V= 4/3 × π × LD × ML × AP
Ellipsoid KV3 formula:
84 + 1.01 x π/24 x LD x (ML + AP)²
Kideny cortex volume =
0.7 x (Total kidney volume – renal sinus volume)
