# Supplementary material for: The number of nephrons in different glomerular diseases
Source: PeerJ. 2019 Sep 4;7:e7640. doi: 10.7717/peerj.7640 (PMC6731770; doi:10.7717/peerj.7640)
Supplement: Supplemental Information 2 [file peerj-07-7640-s002.doc]

**Supplementary Table 1: Comparison of kidney volume, parenchymal volume and cortical volume using different measurement methods. Data represent mean±SD from 35 patients with CT scan available**

| **Methods** | **Total Kidney Volume** (ml) | **Kidney Parenchyma Volume** (ml) | **Kidney Cortical Volume** (ml) |
| --- | --- | --- | --- |
| Cavalieri's method on CT Scan | 280±78 | 182±71 | 133±39 |
| Ellipsoid on US (ml) | 184±87 | 155±73 | 108±73 |
| Ellipsoid-kv3 on US (ml) | 270±88 | 175±82 | 122±66 |
