# Supplementary material for: The number of nephrons in different glomerular diseases
Source: PeerJ. 2019 Sep 4;7:e7640. doi: 10.7717/peerj.7640 (PMC6731770; doi:10.7717/peerj.7640)
Supplement: Supplemental Information 4 [file peerj-07-7640-s004.doc]

codebook to convert numbers to their respective factors

group_1_nephrotic_2_nephritic: factor 1= proteinuric/nephritic syndrome; Factor 2: nephritic syndrome

gender: 0=females; 1= males

age: in years

Urea: serum urea (mg/dl)

Creatinine: serum creatinine (mg/dl)

uricAcid: serum uric acid (mg/dl)

proteinuria_mg24h: 24h proteinuria (mg/die)

eGFR_CKD_EPI: estimated GFR using CKD-EPI formula (ml/min/1.73m2)

kidney_length: maximum length of the kidney (mm)

kidney_volume: kidney volume (ml)

kidney_parenchyma_volume: volume of kidney parenchyma (without renal sinus) (ml)

Kidney_cortical_volume_ml : (ml)

VglomNSG_mm3_x_micro: mean volume of glomeruli (mm3 x 10^-6^)

NglomNSG: total number of glomeruli
